# Lone Atrial Fibrillation Is Associated With Impaired Left Ventricular Energetics That Persists Despite Successful Catheter Ablation

**DOI:** 10.1161/CIRCULATIONAHA.116.022931

**Published:** 2016-10-10

**Authors:** Rohan S. Wijesurendra, Alexander Liu, Christian Eichhorn, Rina Ariga, Eylem Levelt, William T. Clarke, Christopher T. Rodgers, Theodoros D. Karamitsos, Yaver Bashir, Matthew Ginks, Kim Rajappan, Tim Betts, Vanessa M. Ferreira, Stefan Neubauer, Barbara Casadei

**Affiliations:** From Division of Cardiovascular Medicine, University of Oxford, Oxford, UK (R.S.W., A.L., C.E., R.A., E.L., W.T.C., C.T.R., T.D.K., V.M.F., S.N., B.C.); University of Oxford Centre for Clinical Magnetic Resonance Research, Oxford, UK (R.S.W., A.L., C.E., R.A., E.L., W.T.C., C.T.R., T.D.K., V.M.F., S.N.); and Oxford Heart Centre, Oxford University Hospitals NHS Foundation Trust, Oxford, UK (Y.B., M.G., K.R., T.B.).

**Keywords:** arrhythmias, cardiac, atrial fibrillation, catheter ablation, magnetic resonance imaging, magnetic resonance spectroscopy, ventricular dysfunction, left

## Abstract

Lone atrial fibrillation (AF) may reflect a subclinical cardiomyopathy that persists after sinus rhythm (SR) restoration, providing a substrate for AF recurrence. To test this hypothesis, we investigated the effect of restoring SR by catheter ablation on left ventricular (LV) function and energetics in patients with AF but no significant comorbidities.

**Methods:**

Fifty-three patients with symptomatic paroxysmal or persistent AF and without significant valvular disease, uncontrolled hypertension, coronary artery disease, uncontrolled thyroid disease, systemic inflammatory disease, diabetes mellitus, or obstructive sleep apnea (ie, lone AF) undergoing ablation and 25 matched control subjects in SR were investigated. Magnetic resonance imaging quantified LV ejection fraction (LVEF), peak systolic circumferential strain (PSCS), and left atrial volumes and function, whereas phosphorus-31 magnetic resonance spectroscopy evaluated ventricular energetics (ratio of phosphocreatine to ATP). AF burden was determined before and after ablation by 7-day Holter monitoring; intermittent ECG event monitoring was also undertaken after ablation to investigate for asymptomatic AF recurrence.

**Results:**

Before ablation, both LV function and energetics were significantly impaired in patients compared with control subjects (LVEF, 61% [interquartile range (IQR), 52%–65%] versus 71% [IQR, 69%–73%], *P*<0.001; PSCS, –15% [IQR, –11 to –18%] versus −18% [IQR, –17% to –19%], *P*=0.002; ratio of phosphocreatine to ATP, 1.81±0.35 versus 2.05±0.29, *P*=0.004). As expected, patients also had dilated and impaired left atria compared with control subjects (all *P*<0.001). Early after ablation (1–4 days), LVEF and PSCS improved in patients recovering SR from AF (LVEF, 7.0±10%, *P*=0.005; PSCS, –3.5±4.3%, *P*=0.001) but were unchanged in those in SR during both assessments (both *P*=NS). At 6 to 9 months after ablation, AF burden reduced significantly (from 54% [IQR, 1.5%–100%] to 0% [IQR 0%–0.1%]; *P*<0.001). However, LVEF and PSCS did not improve further (both *P*=NS) and remained impaired compared with control subjects (*P*<0.001 and *P*=0.003, respectively). Similarly, there was no significant improvement in atrial function from before ablation (*P*=NS), and this remained lower than in control subjects (*P*<0.001). The ratio of phosphocreatine to ATP was unaffected by heart rhythm during assessment and AF burden before ablation (both *P*=NS). It was unchanged after ablation (*P*=0.57), remaining lower than in control subjects regardless of both recovery of SR and freedom from recurrent AF (*P*=0.006 and *P*=0.002, respectively).

**Conclusions:**

Patients with lone AF have impaired myocardial energetics and subtle LV dysfunction, which do not normalize after ablation. These findings suggest that AF may be the consequence (rather than the cause) of an occult cardiomyopathy, which persists despite a significant reduction in AF burden after ablation.

## Abstract 2

Supplemental Digital Content is available in the text.

**Editorial, see p 1082**

Atrial fibrillation (AF), the most common sustained clinical arrhythmia, is associated with an increased risk of severe stroke,^1^ myocardial infarction,^2^ and premature death.^3^ The worldwide incidence, prevalence, and age-adjusted mortality from AF are increasing, presenting a rapidly growing public health and economic burden.^4^

Mechanistic studies in animal models of pacing-induced AF indicate that atrial remodeling,^5^ oxidative stress,^6^ and impaired coronary reserve^7^ induced by AF are important in arrhythmia maintenance. However, direct translation of these findings is challenging because human AF often reflects multiple interacting causative factors. Indeed, no unique mechanisms for AF have been identified in patients,^8^ even in the up to one third of cases in which AF occurs in the absence of identifiable underlying cardiovascular disease or other specific cause^9^ (conventionally referred to as *lone* AF, although the use of this term has recently been questioned^10^).

Subtle left ventricular (LV) dysfunction has been observed in patients with AF,^11^ with several studies showing that adverse LV structural remodeling and dysfunction are at least partially reversible after restoration of sinus rhythm (SR) with catheter ablation.^12^ Even when the ventricular rate is well controlled, AF may lead to LV dysfunction by reducing myocardial perfusion reserve, impairing calcium handling, and increasing myocardial oxidative stress and fibrosis.^13^ However, large-scale clinical trials of pharmacological strategies designed to restore SR have failed to show prognostic benefit (eg, reduction in death, stroke, or heart failure hospitalizations) compared with ventricular rate control,^14–16^ whereas the effect of catheter ablation remains to be investigated. These results could reflect limited efficacy and potential toxicity of antiarrhythmic medications; nevertheless, it remains the case that at present there are no randomized, clinical data demonstrating that restoration of SR improves major outcomes in patients with AF, raising the possibility that, at least in patients with lone AF and controlled ventricular rate, the arrhythmia may not be the primary cause of LV dysfunction. Instead, a subclinical cardiomyopathy that persists after restoration of SR may provide a substrate for AF initiation and recurrence and an effect on patient prognosis. Here, we used cardiac magnetic resonance (MR) to serially assess atrial and LV volumes and function in patients with AF before and after catheter ablation. We included both early and late postablation MR assessments to quantify the proportion of any improvement in LV function resulting from short-term changes in hemodynamics versus longer-term beneficial cardiac remodeling. We also used phosphorus-31 MR spectroscopy (^31^P-MRS) to determine myocardial energetics before and after ablation because altered cardiac energy metabolism has been identified as an early marker of cardiomyopathy.^17^

## Methods

This prospective study was undertaken in a single tertiary center. The study protocol was approved by a local Research Ethics Committee, and all subjects gave written informed consent.

### Patient Population

Patients undergoing first-time catheter ablation of symptomatic paroxysmal or persistent AF were screened for eligibility. Individuals with significant valvular disease, uncontrolled arterial hypertension, known coronary artery disease, uncontrolled thyroid disease, systemic inflammatory disease, asthma, diabetes mellitus, or obstructive sleep apnea were not enrolled. Further exclusion criteria were contraindications to cardiac MR (including implanted metallic devices and claustrophobia) or gadolinium administration (estimated glomerular filtration rate <30 mL/min).

Clinical management of patients (including ablation strategy and choice of anticoagulant and antiarrhythmic drugs) was at the discretion of the responsible physician.

### Control Group

Control subjects in SR were recruited via poster advertising. Exclusion criteria were identical to those for patients, except that volunteers with a history of palpitations or arrhythmia were not enrolled. Control subjects were selected to match patients for age and sex.

### Study Protocol

Patients were studied at 3 time points: up to 4 weeks before ablation, early after ablation (up to 4 days from the procedure), and at later follow-up (between 6 and 9 months after ablation). Myocardial energetics was not assessed at the early postablation time point. Control subjects were studied at a single time point.

Definitions of paroxysmal and persistent AF were based on contemporary clinical guidelines.^18^ AF burden in patients with paroxysmal AF was assessed before ablation with a 7-day Holter monitor. After ablation, all patients underwent intermittent ECG event monitoring for ≈3 months after an initial 3-month blanking period. Seven-day Holter monitoring was also undertaken after the last follow-up visit. Because the purpose of these investigations was to investigate asymptomatic recurrence of AF and AF burden, they were not undertaken in patients with ECG-documented recurrence of persistent AF. Patients with a history of persistent AF who had undergone cardioversion to SR before ablation (n=3) were excluded from the calculation of change in AF burden postablation.

Patients were classified on the basis of both rhythm during each study assessment and the presence or absence of recurrent AF after the procedure. For the former, patients were categorized by rhythm at the visit ≤4 weeks before ablation and at both follow-up visits. For example, a patient in AF ≤4 weeks before ablationwho had recovered SR early after ablation but relapsed to AF by the last follow-up visit was classified as AF-SR at the early time point and AF-AF at the last follow-up. Patients with at least 1 of the following were classified as having recurrent postprocedural AF: ECG-documented clinical recurrence after the blanking period, ≥30 seconds^19^ of AF or other atrial arrhythmia (eg, atrial flutter or focal atrial tachycardia) on postablation Holter monitoring, or ≥1 rhythm strip (30 seconds) showing AF or other atrial arrhythmia on intermittent ECG monitoring.

Patients who consented to the study but did not contribute any imaging data (because of claustrophobia [n=2], inability to tolerate MR [n=2], or cancellation of the planned ablation [n=1]) are not included in the tables of demographic information.

### Cardiac MR Protocol and Analysis

MR imaging was performed at 1.5 T (Siemens Avanto, Siemens Healthcare, Erlangen, Germany) or 3 T (TIM Trio, Siemens Healthcare) with a 32-channel phased-array coil with the subject supine. Images were acquired during end expiration to minimize the effects of respiratory motion. Pulse sequence parameters and details of the analysis method are provided in the Methods in the online-only Data Supplement.

Cines were acquired with retrospective gating for patients in SR at the time of the scan and with arrhythmia sorting as a first-choice method for patients in AF at the time of the scan. For the minority of patients in AF in whom acceptable images could not be obtained, prospectively triggered cines were acquired instead. Real-time acquisition sequences were also undertaken for all patients in AF during scanning to allow visual assessment of ejection fraction (EF) and corroborate quantitative volumetric analysis. No cases were identified with discordance between visual assessment of EF on real-time acquisition and the results of blinded quantitative volumetric analysis.

Cine image analysis was conducted offline with cmr42 postprocessing software (version 5.1.1, Circle Cardiovascular Imaging Inc, Calgary, ON, Canada). All data sets were anonymized and placed in a random order for contouring. Contours were placed by an operator not involved with data acquisition who was blinded to clinical status, study time point, and rhythm at the time of scanning. Because MR cine loops reconstruct a single R-R interval, R-R variability is not apparent on viewing the cine, and it is possible to be blinded to the presence of AF (Movie I in the online-only Data Supplement). Left atrial maximal volume (LA_max_) and minimal volume (LA_min_) were determined with the biplane area-length method, as previously described,^20^ and used to calculate total left atrial emptying fraction: LAEF=(LA_max_−LA_min_)/LA_max_.

Strain imaging was performed with a prospectively triggered myocardial tagging sequence, as described previously.^21^

Postprocessing of tagging images was performed with CIM-Tag software (Auckland, New Zealand). Semiautomated analysis was performed by aligning a grid to the myocardial tagging planes in end diastole. End systole was determined visually, and tags were adjusted at each frame through the cardiac cycle to derive peak systolic circumferential strain (PSCS) for the midventricular slice, which is expressed as a percentage change from end diastole. Normal PSCS has been described^22^ as –19±2%; impaired myocardial contractility is shown by a more positive value.

Late gadolinium enhancement (LGE) imaging was acquired in 3 short-axis planes (basal, midventricular, and apical) at ≈8 to 10 minutes after intravenous administration of MR contrast agent (total, 0.13 mmol/kg body weight of gadolinium-DTPA; Dotarem, Guerbet, France). The inversion time was adjusted for optimal nulling of remote normal myocardium. Images were assessed by 2 experienced operators at the time of acquisition, and LGE suspected on short-axis imaging was confirmed with additional long-axis imaging. Quantitative analysis was undertaken with cmr42 postprocessing software (as above) on midventricular slices matching the sites of acquisition of ^31^P-MRS data, by setting a signal intensity threshold at 5 SD above the mean intensity of a reference region of interest placed in a remote area of myocardium with no visual evidence of enhancement.

### ^31^P-MRS Protocol and Analysis

^31^P-MRS was performed at 3 T (TIM Trio, Siemens Healthcare) to obtain the ratio of phosphocreatine to ATP concentration (PCr/ATP) from a voxel in the midventricular septum. Subjects fasted overnight and were placed prone with their heart over the center of the ^31^P heart/liver coil, as described previously.^23,24^ Acquisition time was ≈9 minutes in a non–ECG-gated acquisition. Postprocessing was performed as previously described.^25^

### Echocardiography

Transthoracic echocardiography was undertaken in the left lateral position to determine the ratio of peak early diastolic mitral inflow velocity to spectral tissue Doppler-derived peak early diastolic velocity at the mitral annulus (E/E’) as a marker of LV diastolic function.^26^ These measures were averaged over at least 10 cardiac cycles for patients in AF during imaging. E/E’ values reported are the average of lateral and septal measurements.

### Statistical Analysis

A priori sample size calculation was performed to detect a change in the primary outcome of LVEF after ablation. On the basis of the assumption that patients with AF have an LVEF of 55±10%, we calculated that paired analysis of 45 patients before and after ablation would give >90% power to detect a change in LVEF of at least 5 percentage points (2-sided α=0.05). This number of patients would also allow detection of a change in PCr/ATP ratio after ablation of ≥10% with >90% power (2-sided α=0.05). We recruited ≈60 patients to allow for incomplete follow-up, claustrophobia, and other obstacles to completing the protocol. On the basis of pilot data indicating that the PCr/ATP ratio was 1.84±0.41 in patients with AF (n=10) and 2.12±0.26 in normal subjects (n=8), we calculated that recruitment of ≥20 control subjects and ≥40 patients with AF (1:2 allocation) would give >90% power to detect a 13% reduction in PCr/ATP ratio in patients compared with control subjects.

Statistical analyses were performed with IBM SPSS Statistics for Windows, version 20.0 (IBM Corp, Armonk, NY), GraphPad Prism version 6 (GraphPad Software, San Diego, CA), and G*Power version 3.1.9.2.^27^ Normality of data was assessed with Kolmogorov-Smirnov tests. Normally distributed data were compared by use of *t* tests (paired when appropriate) and 1-way ANOVA. Nonnormally distributed unpaired data were compared by use of the Mann-Whitney *U* test or the Kruskal-Wallis test, and nonnormally distributed paired data were compared with the Wilcoxon signed-rank test or the related-samples Friedman 2-way ANOVA by ranks. The χ^2^ test was used to compare proportions. All tests were 2 tailed, and values of *P*<0.05 (after Bonferroni or equivalent adjustment for multiple comparisons when appropriate) were considered significant. Data are shown as mean±SD or median and interquartile range (IQR).

## Results

**Figure 1.**
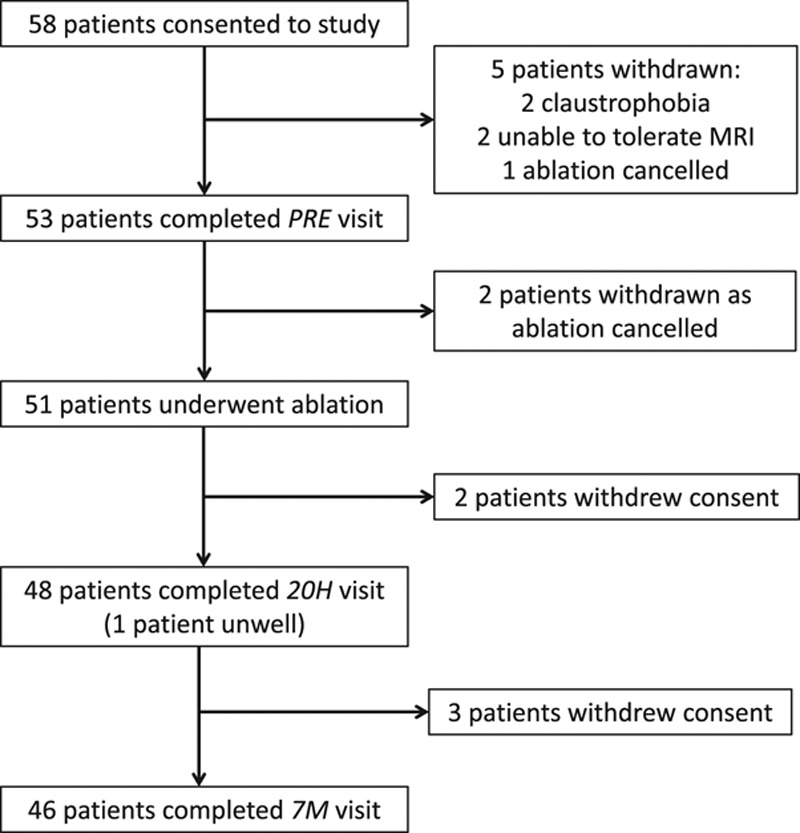
**Flowchart of patients with atrial fibrillation through the study**. The number of patients consenting to the study, completing the preablation (PRE) visit, undergoing ablation, and completing the early (20H) and late (7M) visits is shown. The number of patients not completing each stage is also indicated, with reasons given. MRI indicates magnetic resonance imaging.

A total of 58 patients with AF consented to the study; 53 provided at least 1 set of imaging data, and 45 completed all 3 study visits (Figure 1). The early follow-up visit was conducted at a median of 20 hours after ablation (IQR, 19–23 hours) and the late follow-up visit at a median of 7 months (IQR, 7–9 months).

Baseline characteristics of patients and control subjects, medications, and procedural details of ablation categorized by AF type are summarized in Table 1 and online-only Data Supplement Table I. Median time from the first diagnosis was 3.7 years (IQR, 2.0–7.3 years). Patients with persistent AF had slightly higher body mass index and resting pulse than patients with paroxysmal AF, but exclusion of patients with uncontrolled ventricular rate meant that the resting pulse rate was well controlled in the persistent AF group (median, 72 bpm; IQR, 65–90 bpm). Median AF burden in patients with paroxysmal AF before ablation was 2.6% (≈4.5 h/wk), whereas median AF burden in all patients was 54% (≈91 h/wk).

**Table 1.**
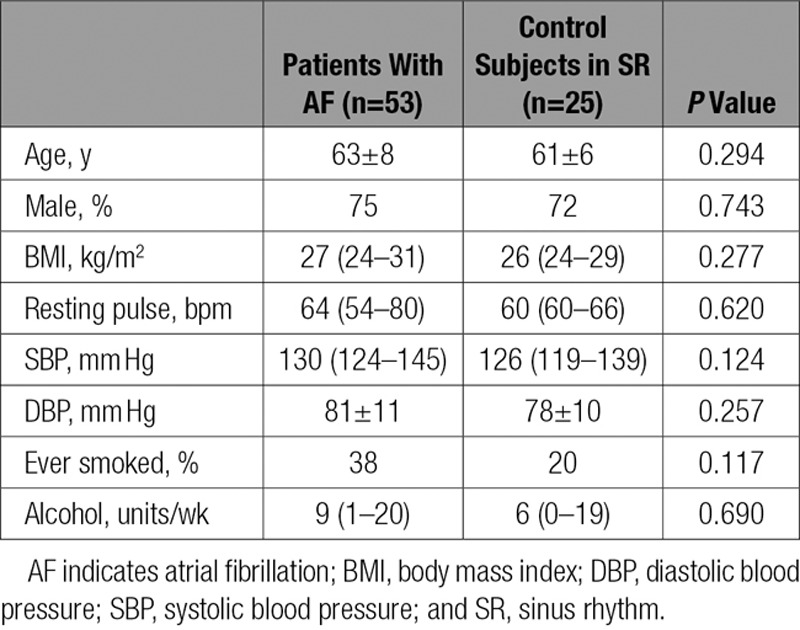
Baseline Characteristics of the Study Groups

### Results in Control Subjects and Patients With AF Before Ablation

LV volume, function, and mass indexes and left atrial volumes and function in control subjects and preablation patients are summarized in Table 2. There were no significant differences in LV end-diastolic volumes between the groups, but patients with AF had significantly larger end-systolic volumes and hence lower LVEF than matched control subjects (both *P*<0.001). However, the impairment in LVEF was subtle, and the median LVEF in patients (61%) fell at the lower end of the normal range by MR imaging in our institution (57%–81%).^28^ PSCS was more clearly abnormal in patients with AF (median, –15% [IQR, –11% to –18%]; normal, –19±2%^22^) and was significantly impaired compared with control subjects (Table 2). As expected, patients with AF had dilated and impaired left atria compared with control subjects (all *P*<0.001; Table 2).

**Table 2.**
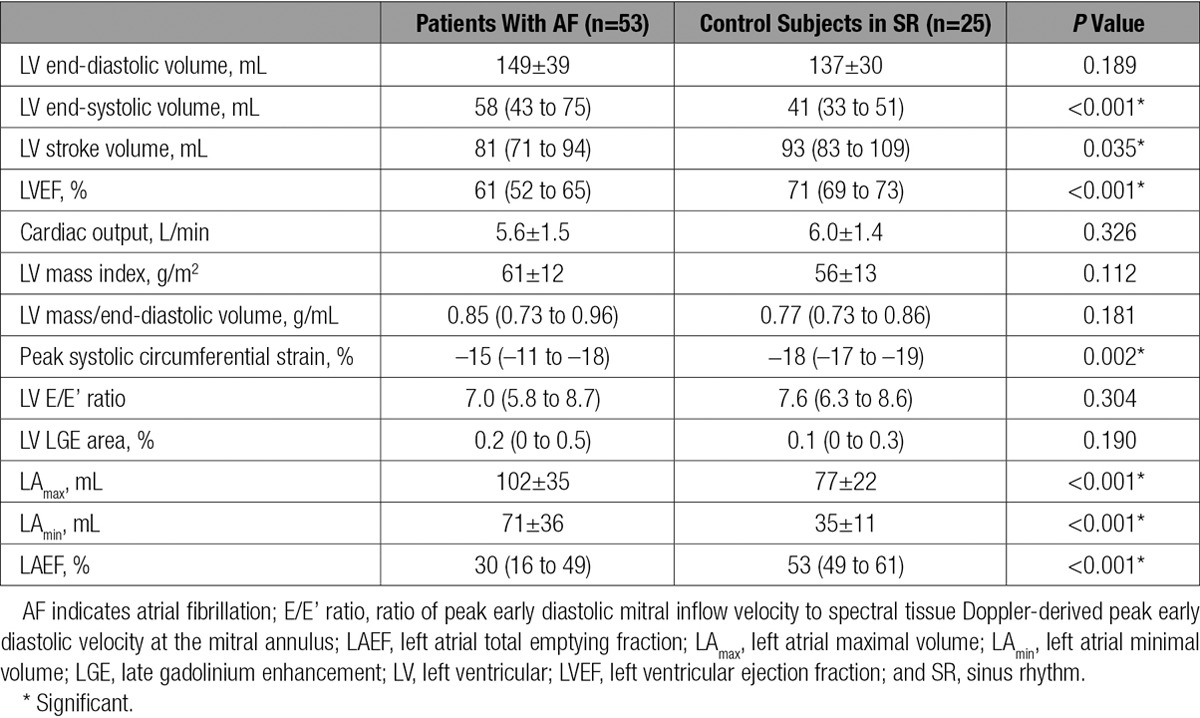
LV and Left Atrial Indexes in the Study Groups

Consistent with our exclusion of patients with uncontrolled hypertension, LV mass index (by MR) and LV diastolic function (by echocardiography) were within the normal range in both patients and control subjects (Table 2). The ratio of LV mass to LV end-diastolic volume (which identifies the presence of concentric LV remodeling in the absence of an absolute increase in LV mass^29^) was also similar between patients and control subjects and was consistent with data reported from healthy control subjects of a similar age in a previous MR study.^30^

**Figure 2.**
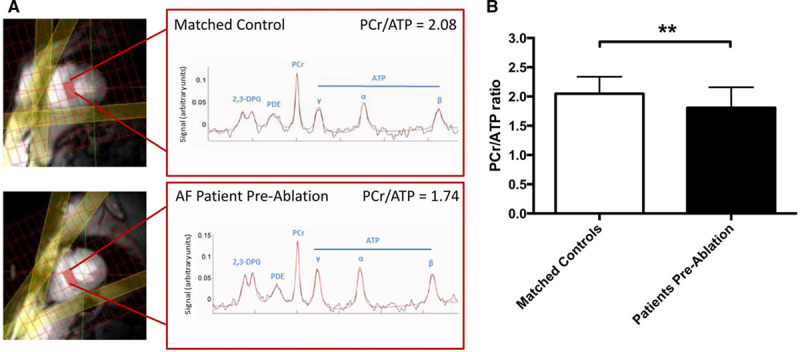
**Representative phosphorus-31 magnetic resonance**(^**31**^**P-MR) spectra and myocardial energetics in the study groups. A**, Images (**left**) show a midventricular short-axis slice, with the selected voxel positioned on the septum and highlighted (red rectangle). Saturation band positions are also shown (yellow). Spectra (**right**) show both raw data (black) and fitted line (red); peaks are labeled corresponding to 2,3-diphosphoglycerate (2,3-DPG), phosphodiester (PDE), phosphocreatine (PCr), and γ, α, and β peaks of ATP. Representative ^31^P-MR spectra from a control subject in SR with a PCr/ATP ratio of 2.08 and a patient with AF before ablation with a PCr/ATP ratio of 1.74. **B**,PCr/ATP ratio in control subjects and patients (n=25 and n=52, respectively; *P*=0.004 by unpaired *t* test).

**Figure 3.**
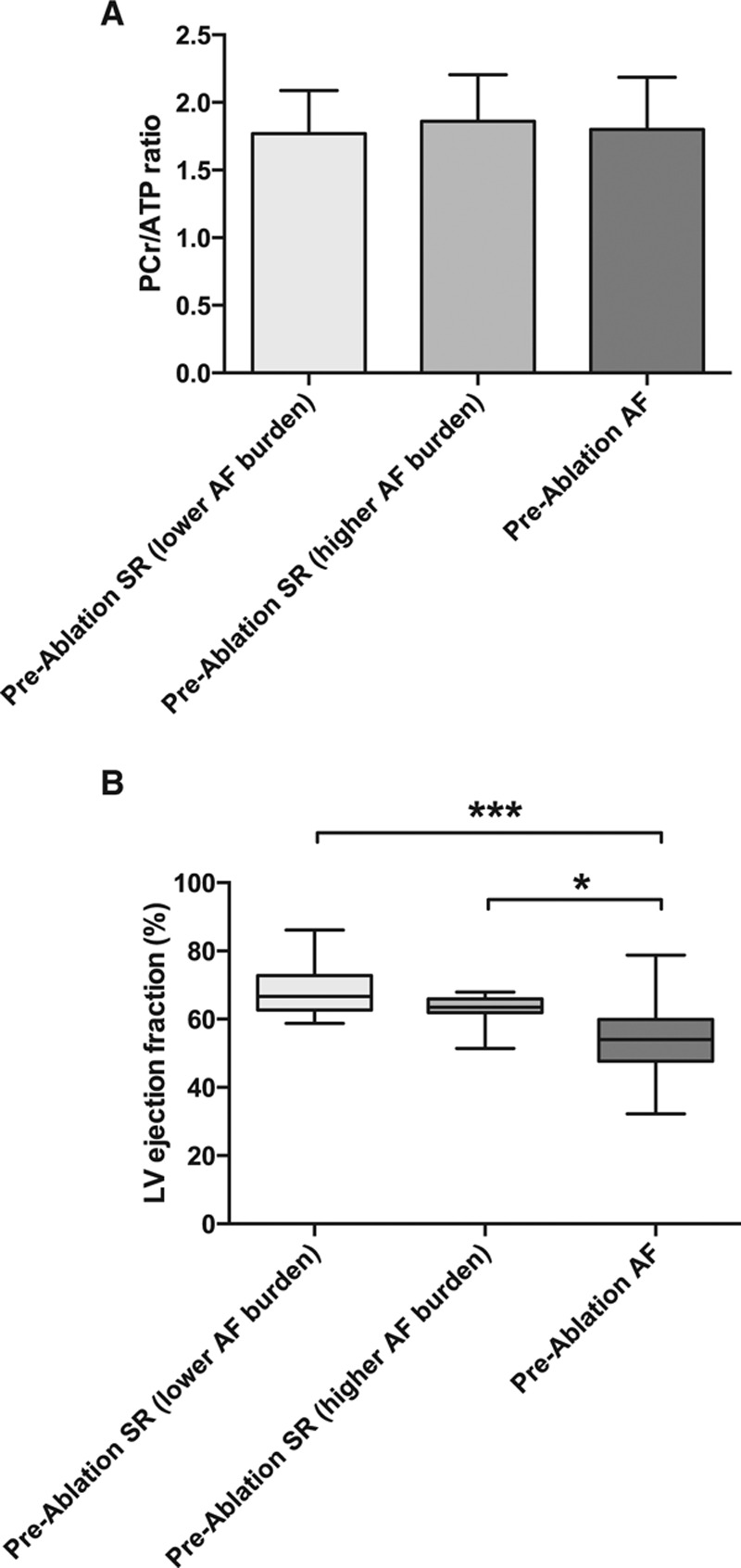
**Effect of intrascan rhythm on left ventricular (LV) ejection fraction**(**EF) and myocardial energetics in patients with atrial fibrillation (AF) before ablation. A**, Myocardial energetics determined by the ratio of phosphocreatine to ATP (PCr/ATP) categorized by rhythm during scan and, for patients in sinus rhythm (SR), by Holter-determined AF burden (higher or lower than median). There is no difference between the groups (n=10 for SR with lower AF burden, n=11 for SR with higher AF burden, and n=28 for AF; *P*=0.84 by 1-way ANOVA). **B**, LVEF in patients with AF before ablation, similarly categorized. Note the significantly lower EF in those in AF compared with those in SR at the time of the scan, regardless of AF burden (Kruskal-Wallis *P*=0.0002). Smaller *P* values stratified by size (**P<0.01, ***P<0.001, ****P<0.0001).

The quality of ^31^P-MRS data was equally good in patients and control subjects (median Cramér-Rao lower bounds^31^ coefficient of variation of PCr/ATP, 15% for patients and 14% for control subjects; *P*=0.52; representative spectra are shown in Figure 2A). Myocardial energetics was significantly impaired in patients with AF compared with control subjects (PCr/ATP, 1.81±0.35 versus 2.05±0.29; *P*=0.004; Figure 2B). Energetics was similarly impaired regardless of the preablation intrascan rhythm and regardless of Holter-determined AF burden for those in SR (PCr/ATP, 1.80±0.39 for preablation AF, 1.86±0.35 for preablation SR with higher-than-median AF burden, and 1.77±0.32 for preablation SR with lower-than-median AF burden; *P*=0.84; Figure 3A). In contrast, presence of AF (rather than SR) during the preablation scan was associated with a significantly lower LVEF (median, 54% [IQR, 48%–60%] versus median 64% [IQR, 63%–69%]; *P*<0.001), but there was no difference in LVEF between patients in SR with higher and those with lower AF burden (*P*=0.34; Figure 3B).

LGE, indicating LV fibrosis or scar, was an infrequent finding that was detected in 8 patients (15%) and 2 control subjects (8%). In 5 patients, LGE had a localized subendocardial or transmural pattern consistent with a small infarct (which had not been identified on echocardiography); 4 of these patients had no obstructive coronary artery disease at angiography, and the cause of infarction was presumed to be embolic. The other 3 patients and both control subjects had a nonischemic pattern of diffuse or patchy fibrosis. No subject had LGE affecting the midventricular septum (ie, the site of sampling for ^31^P-MRS). When the subjects with LGE were removed from the analysis, PCr/ATP and LVEF in patients with AF remained significantly impaired compared with control subjects in SR (PCr/ATP, 1.81±0.37 in the 45 remaining patients versus 2.01±0.26 in the 23 remaining control subjects, *P*=0.02; median LVEF, 61% [IQR, 52%–65%] in the 45 remaining patients versus median 71% [IQR, 69%–73%] in the 23 remaining control subjects, *P*<0.001). Quantitative analysis of LGE demonstrated no difference in the area of enhancement between patients and control subjects (median, 0.2% [IQR, 0%–0.5%] in patients versus 0.1% [IQR, 0%–0.3%] in control subjects; *P*=0.19). No new areas of LGE were noted at the postablation scans.

### Outcomes After Ablation

Ablation was undertaken in 51 patients, with no significant early procedural complications. Radiofrequency ablation was used in 35 patients (69%), cryoballoon ablation in 14 patients (27%), and laser balloon ablation in 2 patients (4%). In the time span between the end of the 3-month blanking period and the 7-month visit, 9 patients (18%) underwent an attempt at electric cardioversion and 3 patients (6%) underwent a second ablation procedure as a result of recurrence of AF or focal left atrial tachycardia.

At 20 hours, the classification of patients by rhythm groups was as follows: SR-SR, 24; AF-SR, 21; and AF-AF, 3. At 7 months, the numbers were the following: SR-SR, 21; AF-SR, 19; AF-AF, 4; and SR-AF, 2. Of 46 patients scanned at 7 months, 25 (54%) had evidence of ≥1 episodes of recurrent AF after ablation. However, Holter-determined AF burden at 7 months was significantly lower than before ablation (median, 0% [IQR, 0%–0.1%] versus 54% [IQR, 1.5%–100%]; *P*<0.001).

### Early Effect of Ablation on LV Function

**Figure 4.**
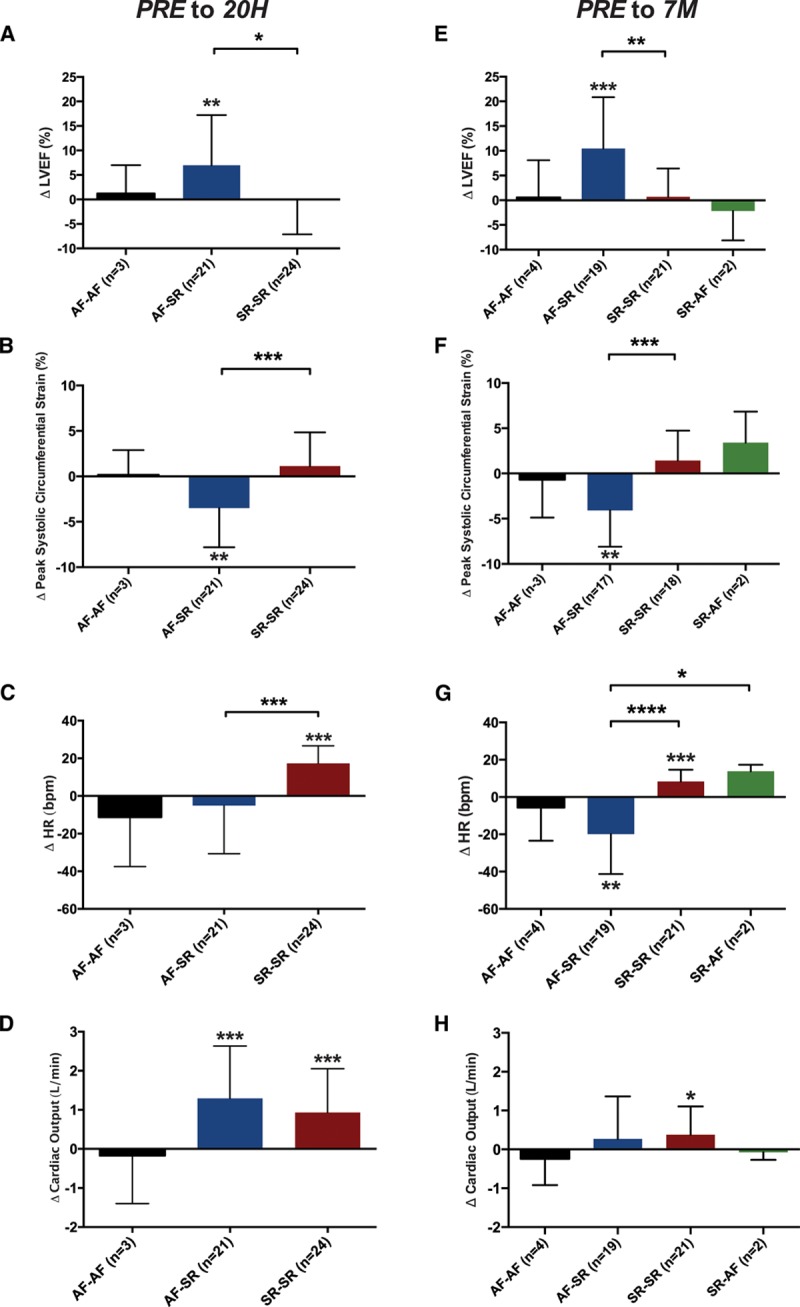
**Change in left ventricular (LV) ejection fraction**(**EF), peak systolic circumferential strain (PSCS), heart rate (HR), and cardiac output early and late after ablation, categorized by the intrascan rhythm at each time point.** One-sample *t* tests assessed whether changes within each subgroup are significantly different from zero. Changes between subgroups were compared by use of 1-way ANOVA; *P* values for subgroup comparisons are Bonferroni corrected for multiple comparisons. **A**, At 20 hours (20H), LVEF improves only in the atrial fibrillation (AF)–sinus rhythm (SR) subgroup (*P*=0.005). **B**, Similarly, PSCS also improves only in the AF-SR subgroup (denoted by a more negative change; *P*=0.001). **C**, Only the SR-SR subgroup shows a significant increase in HR early after ablation (*P*<0.001), and both the AF-SR and SR-SR subgroups show a significant increase in cardiac output, without differences between subgroups (**D**). At 7 months (7M), the pattern of change in LV function from ≤4 weeks before ablation (PRE) in subgroups is similar to that at 20 hours, with the AF-SR subgroup alone showing significant improvement in LVEF (**E**; *P*<0.001) and PSCS (denoted by a more negative change; **F**; *P*=0.001). **G**, The changes in HR from ≤4 weeks before ablationin the AF-SR and SR-SR groups are significantly different (*P*<0.0001), and there is a significant difference between the AF-SR and SR-AF groups (*P*=0.03). **H**, Similar to 20 hours, there are no significant differences between subgroups in the change in cardiac output, with only the SR-SR subgroup demonstrating a small increase (*P*=0.03). Smaller P values stratified by size (**P<0.01, ***P<0.001, ****P<0.0001).

Early after ablation, there was no significant overall change in LVEF (median, 61% [IQR 51%–65%] ≤4 weeks before ablation versus 61% [IQR, 57%–66%] at 20 hours; n=48; *P*=0.07). However, there was a significant increase in LVEF (7.0±10%) in the AF-SR subgroup, unlike the SR-SR and AF-AF subgroups, in which LVEF was unchanged (Figure 4A). A similar pattern was seen for PSCS, with a significant change of –3.5±4.3% (indicating improvement) in the AF-SR subgroup but no change in the other subgroups (Figure 4B).

The SR-SR subgroup showed a significant increase in heart rate from ≤4 weeks before ablation to 20 hours (Figure 4C), consistent with the expected inflammation^32^ and increase in sympathetic activity^33^ induced by the procedure; this effect was entirely counteracted in the AF-SR subgroup by the reduction in ventricular rate associated with recovery of SR.

Overall, cardiac output increased significantly after ablation (6.6±1.6 L/min at 20 hours compared with 5.6±1.5 L/min ≤4 weeks before ablation; *P*<0.001), driven by both the AF-SR and SR-SR subgroups (Figure 4D).

### Late Effect of Ablation on LV Function, Myocardial Energetics, and Left Atrial Indexes

Late after ablation, there was a modest but statistically significant increase in LVEF from before ablation (median, 62% [IQR, 52%–65%] ≤4 weeks before ablation versus 65% [IQR, 59%–68%] at 7 months; n=46; *P*=0.004). However, there was no significant change in LVEF from 20 hours to 7 months (*P*=0.24), and LVEF at 7 months remained lower than in matched control subjects (*P*<0.001), including when the analysis was restricted to patients in SR at 7 months (median, 66% [IQR, 61%–69%] versus 71% [IQR, 69%–73%]; *P*=0.002). Similarly, PSCS in patients in SR at 7 months was impaired compared with matched control subjects (median, –16% versus –18%; *P*=0.035) with no significant overall improvement compared with ≤4 weeks before ablation (*P*=0.375).

The AF-SR subgroup again showed significant improvements in both LVEF and PSCS at 7 months, with no changes seen in the other subgroups (Figures 4E and 4F, respectively). In the 16 patients who were in AF ≤4 weeks before ablation, recovered SR at 20 hours, and remained in SR at 7 months, there was a significant improvement in LVEF across the 3 visits (median, 55% [IQR, 47%–61%] ≤4 weeks before ablation, 60% [IQR, 55%–62%] at 20 hours, and 64% [IQR, 60%–69%] at 7 months; overall trend *P*=0.002; *P*=0.001 for comparison between ≤4 weeks before ablation and 7 months; *P*=0.07 for comparison between 20 hours and 7 months).

At 7 months, the AF-SR subgroup showed a significant reduction in heart rate, whereas the SR-SR subgroup showed a significant increase in heart rate, likely due to withdrawal of rate-controlling medications (Figure 4G). There was a trend toward an overall increase in cardiac output from before ablation(5.9±1.4 L/min at 7 months compared with 5.6±1.5 at ≤4 weeks before ablation; *P*=0.054); with no significant differences between subgroups (Figure 4H).

When patients were grouped by the presence or absence of AF after ablation (rather than by intrascan rhythm), the changes in both LVEF and PSCS from ≤4 weeks before ablation to 7 months were similar between groups (median, 2.7% [IQR, –2.3% to 9.7%] in those with recurrent AF [n=25] versus 3.3% [IQR, –1.4% to 8.3%] in those without recurrent AF [n=21], *P*=0.83 for LVEF; mean –0.2±4% in those with recurrent AF [n=23] versus –2.0±5% in those without recurrent AF [n=17], *P*=0.21 for PSCS).

**Figure 5.**
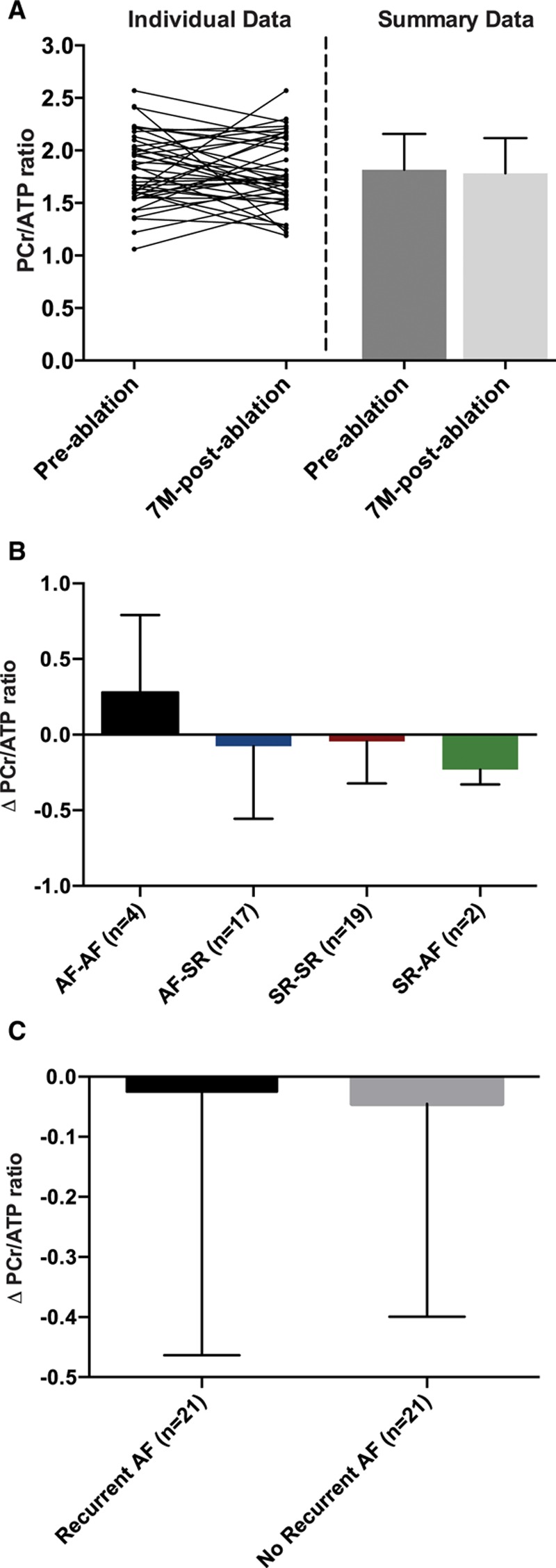
**Myocardial energetics before and late after ablation. A**, In all patients, there was no change in the ratio of phosphocreatine to ATP (PCr/ATP) from ≤4 weeks before to 7 months (7M) after ablation(n=42, *P*=0.57, paired *t* test). There were also no significant differences in the change in PCr/ATP ratio after ablation in any subgroups on the basis of either intrascan rhythm combinations (*P*=0.37, 1-way ANOVA; **B**) or the presence or absence of recurrent atrial fibrillation (AF) after ablation (*P*=0.87, unpaired *t* test; **C**). SR indicates sinus rhythm.

Myocardial energetics, as determined by PCr/ATP ratio, was unchanged from ≤4 weeks before ablationto 7 months (Figure 5A). There were no significant changes in PCr/ATP ratio from ≤4 weeks before ablationto 7 months in any of the intrascan rhythm subgroups (Figure 5B) or between patients with and without AF recurrence after ablation (Figure 5C). Overall, energetics remained impaired at 7 months compared with matched control subjects (1.78±0.33 versus 2.04±0.29; *P*=0.001).

Ablation led to a significant reduction in atrial volume (LA_max_, 86±30 mL at 7 months from 102±37 mL at ≤4 weeks before ablation; n=46 in paired analysis; *P*<0.001), driven by both the AF-SR and SR-SR subgroups (*P*=0.007 and *P*<0.001, respectively; online-only Data Supplement Figure I). Indeed, at 7 months after ablation, atrial volume in patients with AF was not different from that in control subjects (LA_max_, 86±30 mL in patients versus 77±22 mL in control subjects; *P*=0.202). Although LAEF at 7 months improved as expected in the AF-SR subgroup (*P*<0.001; online-only Data Supplement Figure I), there was no significant overall improvement in atrial function postablation (median LAEF, 40% [IQR, 27%–47%] at 7 months from 35% [IQR, 17%–49%] ≤4 weeks before ablation; n=46 in paired analysis; *P*=0.373), and it remained impaired at 7 months compared with control subjects (*P*<0.001). Similarly, LAEF failed to improve even in patients with no recurrent AF (median LAEF, 40% [IQR, 32%–48%] at 7 months from 42% [IQR, 16%–49%] ≤4 weeks before ablation; n=21 in paired analysis; *P*=0.230), again remaining impaired at 7 months compared with control subjects (*P*<0.001).

### Effect of Medications

There were no associations between β-blocker or angiotensin-converting enzyme inhibitor/angiotensin II receptor blocker use (online-only Data Supplement Table I) and myocardial energetics, LVEF, or PSCS either ≤4 weeks before ablationor at 7 months (all *P*=NS).

## Discussion

We undertook a prospective study of patients undergoing first-time ablation of AF, using MR methods to investigate the effect of reducing AF burden on LV function and energetics. For the first time, we demonstrate evidence of significantly impaired myocardial energetics in the ventricular myocardium of patients with lone AF compared with matched control subjects in SR. We also document a subtle reduction in LV systolic function in patients with AF compared with control subjects, with modest improvement (but not normalization) after ablation. Much of the improvement in LV function occurs early after the procedure, is limited to patients with AF who recover SR at the time of the assessment, and thus likely reflects changes in hemodynamics at the time of the scan rather than true beneficial cardiac remodeling resulting from the reduction in AF burden. Indeed, despite a significant reduction in AF burden at 7 to 9 months after ablation, myocardial energetics does not change and LV function does not improve further, remaining impaired compared with control subjects in SR. Taken together, these data imply that lone AF may be the consequence (rather than the cause) of an occult cardiomyopathic process.

### LV Function in AF

AF has been implicated as a cause of LV dysfunction because previous studies have shown an improvement in LV function after catheter ablation^12^ (including reversal of subtle systolic^11^ and diastolic dysfunction^34^). Our patient population had normal LV diastolic function overall, although LVEF by cardiac MR was at the lower end of the normal range at our institution (Table 2), even after successful ablation. There was no evidence of LV dilatation, hypertrophy, or concentric remodeling in our patients with AF compared with matched control subjects in SR, and LGE (indicating LV fibrosis/scar) was an infrequent finding in both groups. These results are consistent with our intention to focus on patients with lone AF and our decision to exclude patients with significant cardiovascular comorbidity or uncontrolled ventricular rate. Nevertheless, there was clear evidence of reduced LV systolic function by both LVEF and PSCS in preablation patients compared with matched control subjects, in keeping with the results of a previous study with similar inclusion criteria.^34^

The effect of AF ablation on LV function has been investigated previously, with a recent meta-analysis showing an overall improvement in LVEF of ≈6% (95% CI, 4%–9%), with the largest improvements seen in patients with persistent AF (compared with patients with paroxysmal AF) and those with low LVEF (compared with those with normal LVEF).^12^ By using cardiac MR imaging both early and late after ablation, our study adds significant further insight into the effect of AF on LV function. Our results indicate that approximately half the overall improvement in LVEF observed at 7 months occurs by 20 hours and that improvement is restricted to patients who were in AF at the scan done ≤4 weeks before ablation and in SR at the scans after ablation (rather than those in whom ablation caused a significant reduction in AF burden on ECG monitoring but who happened to be in SR at the time of both scans). Equally important is our finding that LV function remains impaired in patients with AF after ablation compared with matched control subjects, even when considering only those in SR at 7 months and who had experienced a significant reduction in AF burden after the procedure. The modest improvement in LVEF in patients recovering SR from AF may reflect the increase in LAEF, consistent with recovery of coordinated atrial mechanical activity. However, when considering the entire patient cohort, LAEF was unaffected by ablation and remained abnormal despite a significant reduction in AF burden. This finding is consistent with atrial structural and functional remodeling being driven by a process independent of the arrhythmia itself (eg, fibrotic atrial cardiomyopathy).^35,36^

**Figure 6.**
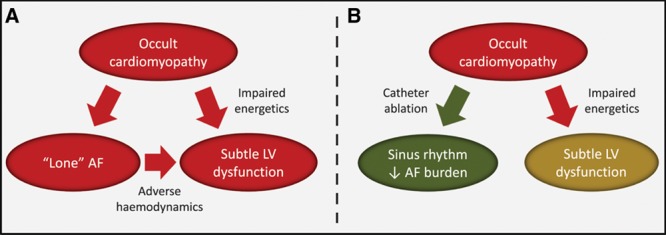
**Proposed schematic representation of the relationships between lone atrial fibrillation (AF), subtle left ventricular (LV) dysfunction, and upstream cardiomyopathy and the effect of ablation. A**, Lone AF and subtle LV dysfunction may be tissue-specific manifestations of an upstream occult cardiomyopathy, characterized by impaired energetics. AF further contributes to LV dysfunction via adverse hemodynamics. **B**, Successful catheter ablation restores sinus rhythm and/or reduces AF burden and leads to a modest increase in LV function via improvement in hemodynamics. However, the underlying cardiomyopathy remains, and myocardial energetics and LV function do not normalize.

Taken together, our data suggest that LV dysfunction in patients with paroxysmal or persistent AF reflects the combination of adverse hemodynamic effects induced acutely by the arrhythmia itself and an underlying cardiomyopathy. Catheter ablation and restoration of SR can improve LV hemodynamics, resulting in modest improvement in LVEF; however, the underlying cardiomyopathy persists, and LV function does not completely normalize (Figure 6).

### Myocardial Energetics in AF

Altered cardiac energy metabolism is an early feature of cardiomyopathy^37^ and is prognostically important.^38,39^ Very few studies have investigated myocardial energetics in AF. Ex vivo investigations have shown a selective reduction in myofibrillar creatine kinase in atrial tissue from patients with AF compared with control subjects in the absence of changes in total creatine kinase or myosin ATPase activity.^40^ In goats with pacing-induced AF, impaired atrial energetics was detected ex vivo shortly after the arrhythmia induction.^41^ We are not aware of any previous in vivo study investigating energetics in the LV myocardium in human AF. We used ^31^P-MRS to noninvasively assess myocardial energetics in patients with AF both before and after ablation. We determined the PCr/ATP ratio, which is reduced when demand for ATP outweighs ATP synthesis (as in ischemia) or with reduction in the total creatine pool (as occurs in heart failure).^17^ Our finding of a significantly lower LV PCr/ATP ratio in patients with AF than in control subjects supports the notion of an underlying cardiomyopathy, particularly in the context of the demonstrated subtle LV dysfunction. It is important to note that the findings that energetics is not affected by heart rhythm at the time of assessment and does not improve after successful AF ablation suggest that the cardiomyopathic process may be “upstream” of AF (Figure 6). This is befitting the idea that underlying atrial disease may actually precede and promote lone AF, as suggested by a number of recent studies.^42–44^

The reduction in PCr/ATP ratio in patients with AF compared with matched control subjects is relatively subtle, which may reflect the relative insensitivity of this measure to the true degree of underlying energetic dysfunction compared with other ^31^P-MRS parameters such as the rate of ATP production (the creatine kinase flux) or the creatine kinase forward rate constant, k_f_.^45^ Future studies should determine the rate of creatine kinase flux in patients with AF because this parameter may reflect both the underlying cardiomyopathy and the response to treatment more accurately than the PCr/ATP ratio.^38,46^

### Clinical Implications and Future Directions

Our results support the notion that lone AF is the consequence of an occult cardiomyopathy that persists despite restoration of SR. This raises the intriguing possibility that such a process (which may develop with aging and exposure to risk factors) may also contribute to recurrence of AF after ablation. However, future studies are needed to examine whether therapeutic strategies that target the adverse cardiometabolic phenotype reduce AF recurrence and improve outcomes. In line with this paradigm, our findings may add mechanistic insight to recent clinical trial data showing that weight loss and intensive risk factor management can dramatically reduce AF burden, symptoms, and adverse cardiac remodeling^47^ and improve AF-free survival after ablation.^48^

We also show that the improvement in LVEF after ablation may not reflect beneficial LV remodeling induced by SR. The results of studies appropriately powered to determine the effects of ablation on hard end points are awaited, and our findings suggest that, at least in lone AF, caution is needed when interpreting improvement in LVEF as a biomarker of possible prognostic benefit from ablation. Further studies are needed to determine whether ^31^P-MRS assessment of myocardial energetics (PCr/ATP or creatine kinase flux) could play an important role in this regard.

### Limitations

AF presents technical challenges to quantitative cardiac MR imaging because of the irregularity of the RR interval. As described in Methods, we have used a number of techniques to counter these issues. In addition, images were analyzed in a blinded fashion and in randomized order to reduce the risk of systematic bias. Although we have not corroborated the quantitative assessments in this study against invasive measures, previous work has shown that MR assessment of volumes and EF is accurate in AF and correlates well with catheterization measurements.^49^

Our study design cannot exclude that 6 to 9 months is too soon for full recovery of ventricular function or energetics after successful ablation; nevertheless, this is a reasonable time point for medium-term follow-up, and failure of normalization by this time is clinically relevant. It is also possible (but in our opinion less likely) that a short duration of lone AF can cause irreversible damage to the ventricular myocardium, with the extent of damage unrelated to cumulative AF burden. This hypothesis could be tested in future population-based, large-scale imaging studies that include rhythm-monitoring investigations.

Last, we studied a specific group of patients with lone AF. Further studies are needed to establish whether our findings are applicable to other AF patient populations.

### Conclusions

Patients with lone AF show impaired LV function and myocardial energetics compared with matched control subjects in SR, and these parameters do not normalize despite a significant reduction in AF burden after successful ablation. These findings imply that AF may be the consequence (rather than the cause) of an underlying cardiomyopathy. Comprehensive therapeutic strategies to target and reverse the adverse cardiometabolic phenotype may be needed to reduce AF recurrence and to improve outcomes.

## Acknowledgments

The authors gratefully acknowledge Judith Delos Santos and Joanne Sellwood for their help and support with patient care.

## Sources of Funding

The study was funded by the British Heart Foundation through a program grant to Dr Casadei (RG/11/15/29375). It was also supported by the National Institute for Health Research Oxford Biomedical Research Center based at Oxford University Hospitals Trust at the University of Oxford, Oxford, United Kingdom. Dr Wijesurendra acknowledges support from the British Heart Foundation Center of Research Excellence, Oxford (RE/08/004). Dr Liu is funded by a British Heart Foundation Clinical Research Training Fellowship (FS/15/11/31233). Dr Rodgers is funded by a Sir Henry Dale Fellowship from the Wellcome Trust and the Royal Society (098436/Z/12/Z).

## Disclosures

None.

## Supplementary Material

## Clinical Perspective

### What Is New?

- Patients with apparently lone atrial fibrillation (AF) have significantly impaired ventricular myocardial energetics, a characteristic and early feature of cardiomyopathy, as well as reduced left ventricular ejection fraction and abnormal peak systolic circumferential strain.
- After catheter ablation, left ventricular function improves rapidly (driven by a switch to sinus rhythm at the time of imaging); however, left ventricular function remains abnormal at 6 to 9 months after ablation (despite a significant reduction in AF burden).
- Myocardial energetics are completely unchanged after ablation, even in patients with substantial and sustained reduction in AF burden.

### What Are the Clinical Implications?

- Our results suggest that apparently lone AF may actually be the consequence (rather than the cause) of an occult cardiomyopathy that is unaffected by ablation.
- Improvement in left ventricular ejection fraction after ablation does not necessarily indicate beneficial cardiac remodeling induced by sinus rhythm; caution may be needed in interpreting improvement in ejection fraction as a biomarker of possible prognostic benefit from ablation.
- Future studies are needed to examine whether therapeutic strategies that target the adverse cardiometabolic phenotype could reduce AF recurrence and improve outcomes.

## References

[1] Lin HJ, Wolf PA, Kelly-Hayes M, Beiser AS, Kase CS, Benjamin EJ, D’Agostino RB (1996). Stroke severity in atrial fibrillation: the Framingham study.. Stroke.

[2] Soliman EZ, Safford MM, Muntner P, Khodneva Y, Dawood FZ, Zakai NA, Thacker EL, Judd S, Howard VJ, Howard G, Herrington DM, Cushman M (2014). Atrial fibrillation and the risk of myocardial infarction.. JAMA Intern Med.

[3] Benjamin EJ, Wolf PA, D’Agostino RB, Silbershatz H, Kannel WB, Levy D (1998). Impact of atrial fibrillation on the risk of death: the Framingham Heart Study.. Circulation.

[4] Chugh SS, Havmoeller R, Narayanan K, Singh D, Rienstra M, Benjamin EJ, Gillum RF, Kim YH, McAnulty JH, Zheng ZJ, Forouzanfar MH, Naghavi M, Mensah GA, Ezzati M, Murray CJ (2014). Worldwide epidemiology of atrial fibrillation: a Global Burden of Disease 2010 Study.. Circulation.

[5] Wijffels MC, Kirchhof CJ, Dorland R, Allessie MA (1995). Atrial fibrillation begets atrial fibrillation: a study in awake chronically instrumented goats.. Circulation.

[6] Carnes CA, Chung MK, Nakayama T, Nakayama H, Baliga RS, Piao S, Kanderian A, Pavia S, Hamlin RL, McCarthy PM, Bauer JA, Van Wagoner DR (2001). Ascorbate attenuates atrial pacing-induced peroxynitrite formation and electrical remodeling and decreases the incidence of postoperative atrial fibrillation.. Circ Res.

[7] Goette A, Bukowska A, Dobrev D, Pfeiffenberger J, Morawietz H, Strugala D, Wiswedel I, Röhl FW, Wolke C, Bergmann S, Bramlage P, Ravens U, Lendeckel U (2009). Acute atrial tachyarrhythmia induces angiotensin II type 1 receptor-mediated oxidative stress and microvascular flow abnormalities in the ventricles.. Eur Heart J.

[8] Schotten U, Verheule S, Kirchhof P, Goette A (2011). Pathophysiological mechanisms of atrial fibrillation: a translational appraisal.. Physiol Rev.

[9] Lévy S (1998). Epidemiology and classification of atrial fibrillation.. J Cardiovasc Electrophysiol.

[10] Wyse DG, Van Gelder IC, Ellinor PT, Go AS, Kalman JM, Narayan SM, Nattel S, Schotten U, Rienstra M (2014). Lone atrial fibrillation: does it exist?. J Am Coll Cardiol.

[11] Tops LF, Den Uijl DW, Delgado V, Marsan NA, Zeppenfeld K, Holman E, van der Wall EE, Schalij MJ, Bax JJ (2009). Long-term improvement in left ventricular strain after successful catheter ablation for atrial fibrillation in patients with preserved left ventricular systolic function.. Circ Arrhythm Electrophysiol.

[12] Zhu P, Zhang Y, Jiang P, Wang Z, Wang J, Yin X, Hou Y (2014). Effects of radiofrequency catheter ablation on left ventricular structure and function in patients with atrial fibrillation: a meta-analysis.. J Interv Card Electrophysiol.

[13] Wijesurendra RS, Casadei B (2015). Atrial fibrillation: effects beyond the atrium?. Cardiovasc Res.

[14] Van Gelder IC, Hagens VE, Bosker HA, Kingma JH, Kamp O, Kingma T, Said SA, Darmanata JI, Timmermans AJ, Tijssen JG, Crijns HJ, Rate Control versus Electrical Cardioversion for Persistent Atrial Fibrillation Study Group (2002). A comparison of rate control and rhythm control in patients with recurrent persistent atrial fibrillation.. N Engl J Med.

[15] Wyse DG, Waldo AL, DiMarco JP, Domanski MJ, Rosenberg Y, Schron EB, Kellen JC, Greene HL, Mickel MC, Dalquist JE, Corley SD, Atrial Fibrillation Follow-up Investigation of Rhythm Management (AFFIRM) Investigators (2002). A comparison of rate control and rhythm control in patients with atrial fibrillation.. N Engl J Med.

[16] Roy D, Talajic M, Nattel S, Wyse DG, Dorian P, Lee KL, Bourassa MG, Arnold JM, Buxton AE, Camm AJ, Connolly SJ, Dubuc M, Ducharme A, Guerra PG, Hohnloser SH, Lambert J, Le Heuzey JY, O’Hara G, Pedersen OD, Rouleau JL, Singh BN, Stevenson LW, Stevenson WG, Thibault B, Waldo AL, Atrial Fibrillation and Congestive Heart Failure Investigators (2008). Rhythm control versus rate control for atrial fibrillation and heart failure.. N Engl J Med.

[17] Hudsmith LE, Neubauer S (2009). Magnetic resonance spectroscopy in myocardial disease.. JACC Cardiovasc Imaging.

[18] Camm AJ, Kirchhof P, Lip GY, Schotten U, Savelieva I, Ernst S, Van Gelder IC, Al-Attar N, Hindricks G, Prendergast B, Heidbuchel H, Alfieri O, Angelini A, Atar D, Colonna P, De Caterina R, De Sutter J, Goette A, Gorenek B, Heldal M, Hohloser SH, Kolh P, Le Heuzey JY, Ponikowski P, Rutten FH, European Heart Rhythm Association, European Association for Cardio-Thoracic Surgery (2010). Guidelines for the management of atrial fibrillation: the Task Force for the Management of Atrial Fibrillation of the European Society of Cardiology (ESC).. Eur Heart J.

[19] Calkins H, Kuck KH, Cappato R, Brugada J, Camm AJ, Chen SA, Crijns HJ, Damiano RJ, Davies DW, DiMarco J, Edgerton J, Ellenbogen K, Ezekowitz MD, Haines DE, Haissaguerre M, Hindricks G, Iesaka Y, Jackman W, Jalife J, Jais P, Kalman J, Keane D, Kim YH, Kirchhof P, Klein G, Kottkamp H, Kumagai K, Lindsay BD, Mansour M, Marchlinski FE, McCarthy PM, Mont JL, Morady F, Nademanee K, Nakagawa H, Natale A, Nattel S, Packer DL, Pappone C, Prystowsky E, Raviele A, Reddy V, Ruskin JN, Shemin RJ, Tsao HM, Wilber D (2012). 2012 HRS/EHRA/ECAS expert consensus statement on catheter and surgical ablation of atrial fibrillation: recommendations for patient selection, procedural techniques, patient management and follow-up, definitions, endpoints, and research trial design.. Europace.

[20] Dodson JA, Neilan TG, Shah RV, Farhad H, Blankstein R, Steigner M, Michaud GF, John R, Abbasi SA, Jerosch-Herold M, Kwong RY (2014). Left atrial passive emptying function determined by cardiac magnetic resonance predicts atrial fibrillation recurrence after pulmonary vein isolation.. Circ Cardiovasc Imaging.

[21] Stuber M, Spiegel MA, Fischer SE, Scheidegger MB, Danias PG, Pedersen EM, Boesiger P (1999). Single breath-hold slice-following CSPAMM myocardial tagging.. MAGMA.

[22] Lawton JS, Cupps BP, Knutsen AK, Ma N, Brady BD, Reynolds LM, Pasque MK (2011). Magnetic resonance imaging detects significant sex differences in human myocardial strain.. Biomed Eng Online.

[23] Tyler DJ, Emmanuel Y, Cochlin LE, Hudsmith LE, Holloway CJ, Neubauer S, Clarke K, Robson MD (2009). Reproducibility of 31P cardiac magnetic resonance spectroscopy at 3 T.. NMR Biomed.

[24] Dass S, Cochlin LE, Holloway CJ, Suttie JJ, Johnson AW, Tyler DJ, Watkins H, Robson MD, Clarke K, Neubauer S (2010). Development and validation of a short 31P cardiac magnetic resonance spectroscopy protocol.. J Cardiovasc Magn Reson.

[25] Rodgers CT, Clarke WT, Snyder C, Vaughan JT, Neubauer S, Robson MD (2014). Human cardiac ^31^P magnetic resonance spectroscopy at 7 Tesla.. Magn Reson Med.

[26] Nagueh SF, Appleton CP, Gillebert TC, Marino PN, Oh JK, Smiseth OA, Waggoner AD, Flachskampf FA, Pellikka PA, Evangelisa A (2009). Recommendations for the evaluation of left ventricular diastolic function by echocardiography.. Eur J Echocardiogr.

[27] Faul F, Erdfelder E, Lang AG, Buchner A (2007). G*Power 3: a flexible statistical power analysis program for the social, behavioral, and biomedical sciences.. Behav Res Methods.

[28] Hudsmith LE, Petersen SE, Francis JM, Robson MD, Neubauer S (2005). Normal human left and right ventricular and left atrial dimensions using steady state free precession magnetic resonance imaging.. J Cardiovasc Magn Reson.

[29] Verdecchia P, Schillaci G, Borgioni C, Ciucci A, Battistelli M, Bartoccini C, Santucci A, Santucci C, Reboldi G, Porcellati C (1995). Adverse prognostic significance of concentric remodeling of the left ventricle in hypertensive patients with normal left ventricular mass.. J Am Coll Cardiol.

[30] Dweck MR, Joshi S, Murigu T, Gulati A, Alpendurada F, Jabbour A, Maceira A, Roussin I, Northridge DB, Kilner PJ, Cook SA, Boon NA, Pepper J, Mohiaddin RH, Newby DE, Pennell DJ, Prasad SK (2012). Left ventricular remodeling and hypertrophy in patients with aortic stenosis: insights from cardiovascular magnetic resonance.. J Cardiovasc Magn Reson.

[31] Cavassila S, Deval S, Huegen C, van Ormondt D, Graveron-Demilly D (2001). Cramér-Rao bounds: an evaluation tool for quantitation.. NMR Biomed.

[32] Lim HS, Schultz C, Dang J, Alasady M, Lau DH, Brooks AG, Wong CX, Roberts-Thomson KC, Young GD, Worthley MI, Sanders P, Willoughby SR (2014). Time course of inflammation, myocardial injury, and prothrombotic response after radiofrequency catheter ablation for atrial fibrillation.. Circ Arrhythm Electrophysiol.

[33] Hsieh MH, Chiou CW, Wen ZC, Wu CH, Tai CT, Tsai CF, Ding YA, Chang MS, Chen SA (1999). Alterations of heart rate variability after radiofrequency catheter ablation of focal atrial fibrillation originating from pulmonary veins.. Circulation.

[34] Reant P, Lafitte S, Jaïs P, Serri K, Weerasooriya R, Hocini M, Pillois X, Clementy J, Haïssaguerre M, Roudaut R (2005). Reverse remodeling of the left cardiac chambers after catheter ablation after 1 year in a series of patients with isolated atrial fibrillation.. Circulation.

[35] Kottkamp H (2012). Fibrotic atrial cardiomyopathy: a specific disease/syndrome supplying substrates for atrial fibrillation, atrial tachycardia, sinus node disease, AV node disease, and thromboembolic complications.. J Cardiovasc Electrophysiol.

[36] Schotten U, Dobrev D, Platonov PG, Kottkamp H, Hindricks G (2016). Current controversies in determining the main mechanisms of atrial fibrillation.. J Intern Med.

[37] Neubauer S (2007). The failing heart: an engine out of fuel.. N Engl J Med.

[38] Bottomley PA, Panjrath GS, Lai S, Hirsch GA, Wu K, Najjar SS, Steinberg A, Gerstenblith G, Weiss RG (2013). Metabolic rates of ATP transfer through creatine kinase (CK Flux) predict clinical heart failure events and death.. Sci Transl Med.

[39] Neubauer S, Horn M, Cramer M, Harre K, Newell JB, Peters W, Pabst T, Ertl G, Hahn D, Ingwall JS, Kochsiek K (1997). Myocardial phosphocreatine-to-ATP ratio is a predictor of mortality in patients with dilated cardiomyopathy.. Circulation.

[40] Mihm MJ, Yu F, Carnes CA, Reiser PJ, McCarthy PM, Van Wagoner DR, Bauer JA (2001). Impaired myofibrillar energetics and oxidative injury during human atrial fibrillation.. Circulation.

[41] Ausma J, Coumans WA, Duimel H, Van der Vusse GJ, Allessie MA, Borgers M (2000). Atrial high energy phosphate content and mitochondrial enzyme activity during chronic atrial fibrillation.. Cardiovasc Res.

[42] McGann C, Akoum N, Patel A, Kholmovski E, Revelo P, Damal K, Wilson B, Cates J, Harrison A, Ranjan R, Burgon NS, Greene T, Kim D, Dibella EV, Parker D, Macleod RS, Marrouche NF (2014). Atrial fibrillation ablation outcome is predicted by left atrial remodeling on MRI.. Circ Arrhythm Electrophysiol.

[43] Konrad T, Theis C, Mollnau H, Sonnenschein S, Ocete BQ, Bock K, Münzel T, Rostock T (2015). Primary persistent atrial fibrillation: a distinct arrhythmia subentity of an ablation population.. J Cardiovasc Electrophysiol.

[44] Kottkamp H (2013). Human atrial fibrillation substrate: towards a specific fibrotic atrial cardiomyopathy.. Eur Heart J.

[45] Smith CS, Bottomley PA, Schulman SP, Gerstenblith G, Weiss RG (2006). Altered creatine kinase adenosine triphosphate kinetics in failing hypertrophied human myocardium.. Circulation.

[46] Hirsch GA, Bottomley PA, Gerstenblith G, Weiss RG (2012). Allopurinol acutely increases adenosine triphosphate energy delivery in failing human hearts.. J Am Coll Cardiol.

[47] Abed HS, Wittert GA, Leong DP, Shirazi MG, Bahrami B, Middeldorp ME, Lorimer MF, Lau DH, Antic NA, Brooks AG, Abhayaratna WP, Kalman JM, Sanders P (2013). Effect of weight reduction and cardiometabolic risk factor management on symptom burden and severity in patients with atrial fibrillation: a randomized clinical trial.. JAMA.

[48] Pathak RK, Middeldorp ME, Lau DH, Mehta AB, Mahajan R, Twomey D, Alasady M, Hanley L, Antic NA, McEvoy RD, Kalman JM, Abhayaratna WP, Sanders P (2014). Aggressive risk factor reduction study for atrial fibrillation and implications for the outcome of ablation: the ARREST-AF cohort study.. J Am Coll Cardiol.

[49] Hundley WG, Meshack BM, Willett DL, Sayad DE, Lange RA, Willard JE, Landau C, Hillis LD, Peshock RM (1996). Comparison of quantitation of left ventricular volume, ejection fraction, and cardiac output in patients with atrial fibrillation by cine magnetic resonance imaging versus invasive measurements.. Am J Cardiol.

