# Supplementary material for: Lone Atrial Fibrillation Is Associated With Impaired Left Ventricular Energetics That Persists Despite Successful Catheter Ablation
Source: Circulation. 2016 Oct 10;134(15):1068–81. doi: 10.1161/CIRCULATIONAHA.116.022931 (PMC5054971; doi:10.1161/CIRCULATIONAHA.116.022931)
Supplement: Supplementary file 2 [file cir-134-1068-s002.pdf]

SUPPLEMENTAL MATERIAL

Methods

Cardiac magnetic resonance cine imaging

Cardiac volumes were acquired using steady state free precession (SSFP) imaging. Scan parameters were typically: voxel size 2.0x2.0x8.0mm, FOV=380x380mm, TR/TE 39.6/1.12ms, flip angle 55°, matrix 192x192, GRAPPA=3, 24 reference lines, segments=15, concatenations=1. Pilot images were initially acquired and used to plan and acquire horizontal long axis (HLA), vertical long axis (VLA), left ventricular outflow tract (LVOT) long axis and short axis stack images.

LV short axis epicardial and endocardial borders were manually contoured at end diastole and end systole. LV end systolic (ESV) and end diastolic (EDV) volumes were used to calculate stroke volume (SV) as SV = EDV-ESV. Ejection fraction (EF) and cardiac output (CO) were calculated as EF = SV/EDV and CO=SV x HR, respectively. LV mass was calculated by subtracting the endocardial volume from the epicardial volume, based on prior knowledge of myocardial specific gravity (1.05 g/cm^3).

Cardiac magnetic resonance tagging

Tagged cine MRIs were acquired with an ECG-triggered segmented k-space gradient echo sequence with spatial modulation of magnetization in orthogonal planes. The scan parameters were typically: voxel size 2.1 x 1.4 x 8.0 mm, FOV = 360 x 292 mm, matrix 141 x 256, TR/TE = 40.45/3.89 ms, flip angle 14°, segments = 9, phases = 16, concatenations = 1, grid tag distance = 7mm, bandwidth = 184 Hz/Px.

Late gadolinium enhancement (LGE) imaging

LGE imaging was acquired using a T1-weighted phase-sensitive inversion recovery sequence. Scan parameters were typically: voxel size 2.0 x 1.5 x 8.0 mm, matrix 144x256, field-of-view=380x285mm, TR/TE=800.20/3.36ms, flip angle 25°, GRAPPA=2, 24 reference lines, segments=25, phases=1, concatenations=1, measurements=1, bandwidth=130Hz/Px.

^{31}P magnetic resonance spectroscopy

A 3-dimensional acquisition-weighted chemical shift imaging technique is used with 10 averages at the centre of k-space and ultrashort echo time (TE) to minimise T2 effects and first-order phase artefacts. Acquisition time is ~9 minutes, and an optimized radiofrequency pulse centred between the γ- and α-ATP resonance frequencies is used to ensure uniform excitation of all spectral peaks. Five Nuclear Overhauser Effect (NOE) pulses (2.5 ms, 222.2 V separated by 80.5 ms) are used to increase signal to noise. Acquisition matrix is 16 x 8 x 8 and field of view is 240 x 240 x 200 mm^3. Three 25-mm-thick saturation bands are used to minimise signal contamination in the heart, 2 placed over chest wall muscle and 1 placed over liver. The chemical shift imaging grid is placed with a central voxel in the mid-ventricular septum and rotated to maximize coverage of the septal myocardium.

The spectrum from the mid-ventricular septal voxel was fitted using a custom implementation of AMARES (the advanced method for accurate, robust, and efficient spectral fitting) in our semi-automated spectroscopy post-processing pipeline[1] in Matlab (Mathworks Inc, Nattick, USA). Fitting used prior knowledge specifying 11 Lorentzian peaks (a,b,g-ATP multiplet components, PCr, PDE, and 2x2,3-DPG) and fixed amplitude ratios

and scalar couplings for the multiplets. The fitted amplitudes were then corrected for blood contamination by subtracting 30% of the average of the two 2,3-DPG signals from each of the ATP amplitudes. The remaining PCr and ATP signals were corrected for the effects of partial saturation using the flip angle at the centre of the voxel, assuming no motion effects and with the T_1 values shown in Table 1 in Rodgers et al[2].

Data

Supplementary Table 1. Baseline Characteristics and Ablation Details for Patients, Categorised by AF Type

	**Paroxysmal AF (n=27)**	**Persistent AF (n=26)**	**p value**
**Male (%)**	78	73	0.691
**BMI (kg/m^2)**	25 (24 – 29)	29 (25 – 35)	**0.033**
**Resting pulse (bpm)**	56 (48 – 64)	72 (65 – 90)	**0.001**
**CHA_2DS_2-VASc**	1 (0 – 2)	2 (1 – 2)	**0.046**
**In AF at *PRE* (%)**	22	88	**<0.001**
Ablation type (%)			
**Radiofrequency**	48	85	**0.005**
**Cryoballoon**	48	4	**<0.001**
**Laser**	4	4	0.978
Medications at PRE (%)			
**ACEI / ARB**	26	62	**0.009**
**Beta-blocker**	19	73	**<0.001**
**Flecainide**	37	8	**0.011**
**Sotalol**	33	8	**0.021**
**Digoxin**	4	27	**0.018**
**Dronedarone**	7	0	0.157
**Amiodarone**	4	12	0.280
**Warfarin**	48	69	0.119
**NOAC**	33	31	0.842
Medications at 7M (%)			
**ACEI / ARB**	30	54	0.074
**Beta-blocker**	15	42	**0.026**
**Flecainide**	19	0	**0.021**
**Sotalol**	11	0	0.080
**Digoxin**	0	4	0.314
**Dronedarone**	7	0	0.157
**Amiodarone**	4	8	0.530
**Warfarin**	41	46	0.691
**NOAC**	19	19	0.947

Footnote: Of 26 persistent AF patients, 3 (12%) underwent cardioversion to SR prior to the pre-ablation CMR scan. Abbreviations: ACEI, angiotensin converting enzyme inhibitor; AF, atrial fibrillation; ARB; angiotensin II receptor antagonist; BMI, body mass index; NOAC; novel oral anticoagulant.

Supplementary Figure 1. Change in Left Atrial Maximal Volume and Emptying Fraction Early and Late after Ablation, Categorised by the Intra-scan Rhythm at each Timepoint.

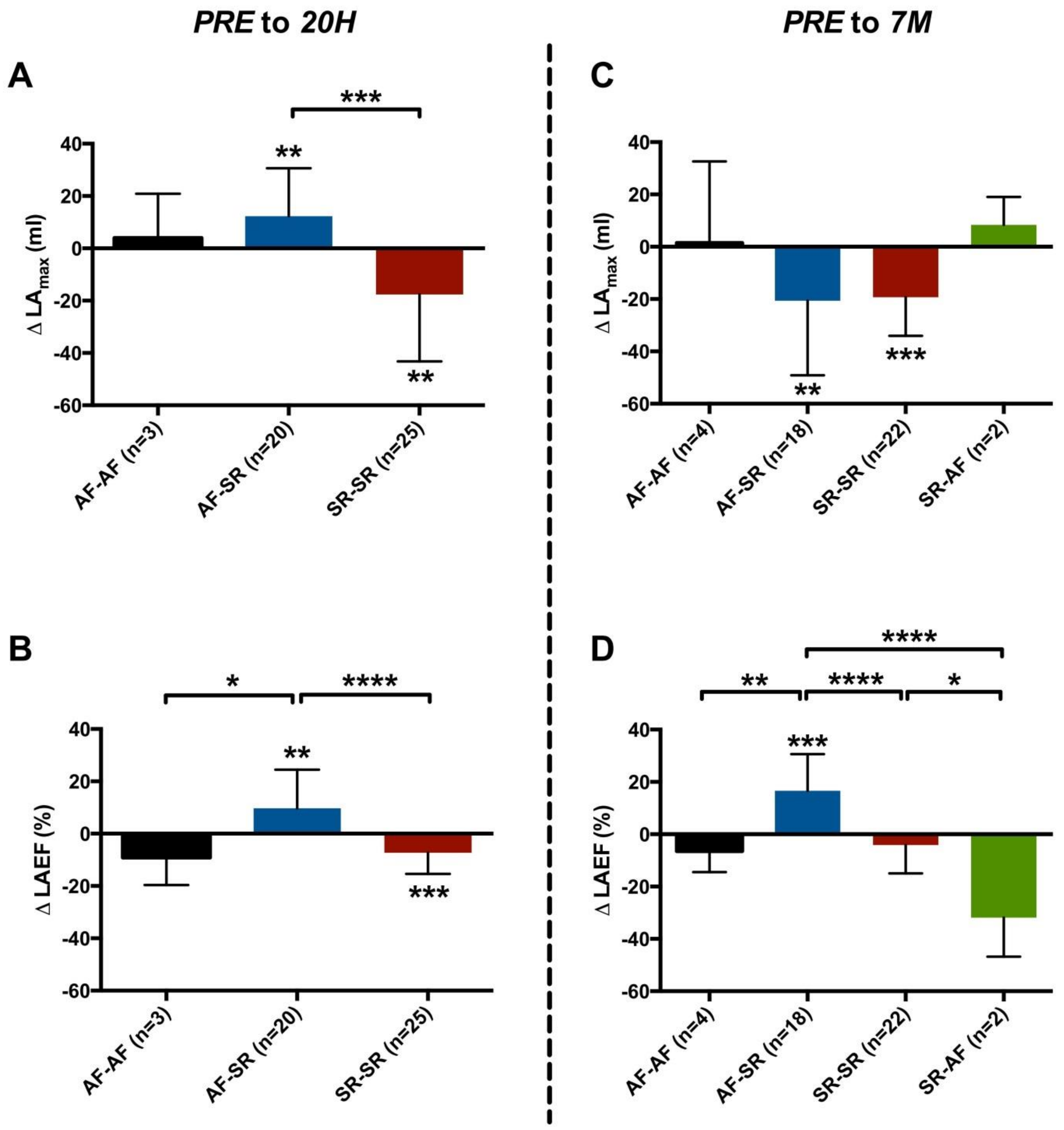

One-sample t-tests assessed if changes within each sub-group are significantly different to zero. Changes between sub-groups were compared using one-way ANOVA; p values for sub-group comparisons are Bonferroni-corrected for multiple comparisons. LA_{max} denotes maximal left atrial volume and LAEF denotes left atrial emptying fraction. (**A**) At *20H*, maximal left atrial volume increases in the AF-SR sub-group (p=0.008) and decreases in the SR-SR sub-group (p=0.002). (**B**) Similarly, left atrial emptying fraction improves in the AF-SR sub-group (p=0.009) and worsens in the SR-SR sub-group at *20H* (p<0.001). (**C**) At *7M,* both the AF-SR and SR-SR sub-groups show decreases in maximal left atrial volume (p=0.007 and p<0.001, respectively). (**D**) Only the AF-SR sub-group show a significant improvement in left atrial emptying fraction at *7M* (p<0.001).

Supplementary Movie 1. Representative Short Axis Cine Stack from a Patient in Atrial Fibrillation during Acquisition.

Supplementary References

1. Purvis LAB CW, Biasiolli L, Robson MD, Rodgers CT. Linewidth constraints in Matlab AMARES using per-metabolite T2 and per-voxel ΔB0. In Proceedings of the 22nd Annual Meeting of ISMRM-ESMRMB, Milan, Italy, 2014. Abstract 2885. 2014.
2. Rodgers CT, Clarke WT, Snyder C, Vaughan JT, Neubauer S, Robson MD. Human cardiac ^{31}P magnetic resonance spectroscopy at 7 tesla. Magnetic Resonance in Medicine 2014;**72**:304-315.
